# Characterization and Co-Adsorption Mechanism of Magnetic Clay-Biochar Composite for De-Risking Cd(II) and Methyl Orange Contaminated Water

**DOI:** 10.3390/ijms24065755

**Published:** 2023-03-17

**Authors:** Fengxiao Zhao, Rui Shan, Shuang Li, Haoran Yuan, Yong Chen

**Affiliations:** 1 Guangzhou Institute of Energy Conversion, Chinese Academy of Sciences, Guangzhou 510640, China; 2 School of Energy Science and Engineering, University of Science and Technology of China, Guangzhou 510640, China; 3 Xiamen Key Laboratory of Clean and High-Valued Utilization for Biomass, Fujian Engineering and Research Center of Clean and High-Valued Technologies for Biomass, College of Energy, Xiamen University, Xiamen 361102, China; 4 CAS Key Laboratory of Renewable Energy, Guangdong Provincial Key Laboratory of New and Renewable Energy Research and Development, Guangzhou 510640, China

**Keywords:** co-adsorption, clay-biochar composite, Cd(II), methyl orange

## Abstract

The application of the adsorption method in sewage treatment has recently become a hot spot. A novel magnetic clay-biochar composite (BNT-MBC) was fabricated by co-pyrolysis of bentonite and biomass after being impregnated with Fe (NO_3_)_3_·9H_2_O. Its adsorption capacity for Cd(II) and methyl orange was approximately doubled, reaching a maximum of 26.22 and 63.34 mg/g, and could be easily separated from the solution by using external magnets with its saturation magnetization of 9.71 emu/g. A series of characterizations including surface morphology and pore structure, elemental analysis, functional group analysis and graphitization were carried out, showing that the specific surface area was increased 50 times by loading 20 wt.% bentonite, while its graphitization and oxygen-containing functional groups were also enhanced. The isotherm fitting indicated that Cd(II) was adsorbed in multiple layers, while methyl orange was in both monolayer and multilayer adsorptions. The kinetic fitting indicated that chemisorption was the rate-limiting step of both, and it was also a complex process controlled by two steps with the fitting of intra-particle diffusion. In the binary system of Cd(II) and methyl orange, the co-existing pollutants facilitated the adsorption of the original one, and there was no competition between adsorption sites of Cd(II) and methyl orange. BNT-MBC also exhibited good reusability and can be magnetically recovered for recycling. Thus, the magnetic clay-biochar composite BNT-MBC is a cost-effective and promising adsorbent for simultaneous removing Cd(II) and methyl orange from wastewater.

## 1. Introduction

The textile dyeing and printing industry has been currently one of the world’s largest polluters and consumers of water resources [1,2]. Globally, the annual production of dyes has exceeded one million tons, where 100–200 tons of water would be consumed when processing one ton of textiles, and 80–90% of the discharged wastewater was organic dye wastewater [3,4,5]. 

Among them, the use of azo dyes reaches more than two-thirds of the total dyes, and most of its organic components are aromatic and heterocyclic compounds with chromogenic groups (such as -N=N-, -N=O) or polar groups (such as -SO_3_Na, -OH, -NH_2_) [5,6]. The azo dye wastewater is chemically inert, complex in composition, and highly colored [7,8]. The flow of highly colored dye wastewater into ecological watersheds will reduce the transparency of water bodies and seriously affect the growth of organisms in the environment [9,10]. In addition, the affinity between some colorants and fibers is low, so mordants containing heavy metals (HMs) are often used in the traditional fixation process to improve the color fastness of dyed fabrics [5,11,12]; the coexistence of organic pollutants and HMs is also frequently reported in livestock wastewater [13]. HM pollutants are characterized by accumulation, wide sources, easy enrichment transfer along the food chain, as well as difficulty in degradation [14,15]. In some cases, HMs may react with organics coexisting in water bodies to produce metal organic pollutants with stronger toxicity [16,17]. In-depth exposure to wastewater with the coexistence of organic pollutants and heavy metals could also lead to malignant mutation of other organisms [18]. Therefore, the complexity of the actual effluent should not be underestimated for water treatment.

Adsorption has recently come to the forefront of environmental remediation for simplicity, lack of chemical involvement, and low energy consumption [19,20,21]. And pyrolysis of biomass, such as agricultural and forestry waste, could be applied to the manufacture of adsorbents to effectively adsorb hazardous substances from wastewater [22,23]. Many modification processes during pyrolysis (e.g., chemical and physical activation techniques) have been reported to effectively enhance the pore structure of biochar, improve the adsorption sites, and ultimately obtain new adsorbent materials with optical, electrical, and magnetic properties [24,25]. In recent years, some experimental studies have been conducted in China and abroad on the co-pyrolysis of biochar and clay minerals with low cost and easy access to gain the improvement of its adsorption capacity [26,27].

Bentonite is a hydrated aluminosilicate or aluminosilicate mineral, mainly composed of montmorillonite with the chemical formula (Al, Mg)_2_·(OH)_2_ (Si, Al)_4_O_10_(Ca)_x_·nH_2_O [28,29]; its crystal structure belongs to the monoclinic crystal system and consists of structural unit layers and interlayer domains. Bentonite is easy to mine, nontoxic, and inexpensive. It also has lamellar structures, intercalation property, and relatively high surface area. Furthermore, the cations (Ca^2+^, Na^2+^, Mg^2+^) present in its interlayer space can be exchanged with positively charged pollutants through an ion exchange mechanism, which makes it a potential adsorbent. However, due to their small particle size, clays are difficult to separate from the liquid phase and cannot be used as fixed bed media in water treatment [30]. Instead, co-pyrolysis of biochar matrix and bentonite particles on its surface is proved to be an economical and feasible method to solve this problem.

Gao et al. [30] prepared clay-loaded biochar adsorbent by mixing montmorillonite and kaolinite with biomass, then pyrolyzing them under 600 °C for 60 min. It was found that the aromatic structure of the material was strengthened, with the methylene blue adsorption capacity raising from 11 to 110 mg _MB_/g _char_ after modification and pyrolysis, and the main adsorption mechanism was hydrogen bonding and pore filling. A series of engineered biochar was developed by Ramola et al. [31], among which the bentonite biochar prepared at 700 °C was effective in removing Pb(II) (99.9%), where bentonite acted as a catalyst on biochar surface, improving its yield, pH, surface morphology, functional groups, etc. The adsorption process was greatly influenced by the electrostatic attraction between Pb(II) and mineral groups. In the field of removing pesticide residues from the soil, montmorillonite-modified walnut shell biochar [32] also exhibited good adsorption performance on iprodione, and the analysis showed that hydrogen bonding, π-π bonding, ligand bonding, and hydrophobic interactions are the key adsorption mechanisms. All these studies indicated that the composite prepared from clay and biochar could be used as an effective adsorbent for removing organic or HM contaminants from the liquid phase. In addition to co-pyrolysis of biomass and clay materials, Yu et al. [33] proposed a new strategy of co-pyrolysis of biomass and clay mediated by trace metal elements using microwave-assisted method, which showed better stability and phosphorus leaching resistance in the field of soil remediation.

However, most studies on clay minerals-biochar composite adsorbents have targeted single pollutants, and little consideration has been given to the treatment of complex systems where organic dyes and HMs coexist, and the adsorption behavior of organic dyes and HMs on clay mineral-biochar composites remains unclear [27,32,34]. In addition, the recovery methods of the adsorbents after use have not been focused on. Several studies have shown that magnetic biochar can partially improve the adsorption capacity of its precursor biochar and can be easily separated from solution with the use of external magnets [35,36]. Therefore, our group reports a simple and effective synthesis of magnetic clay biochar composites via co-pyrolysis of bentonite with Fe (NO_3_)_3_·9H_2_O impregnated corn straw, and the adsorption performance of this material on methyl orange (MO) and Cd(II) was investigated. The biochar provides a porous structure to support clay micron particles and nanoparticles as a carbon-containing matrix, while the impregnation of Fe (NO_3_)_3_·9H_2_O provides magnetic properties. In this study, MO was selected as the representative of organic dye pollutants, and Cd(II) was selected as the representative of HM pollutants to simulate the complexity of realistic wastewater, while we explored the adsorption with SEM-EDS, FT-IR, N_2_ adsorption–desorption characterization, kinetic, and adsorption isotherm fitting to investigate the mechanism of the process. Meanwhile, adsorption–desorption experiments were conducted to investigate its recycling performance, which makes the study of clay minerals-biochar composite more practical in the field of environmental remediation.

## 2. Results and Discussion

### 2.1. Adsorbent Characteriazation

For the purpose of verifying the hypothesis that clay minerals can improve the adsorption capacity of biochar when co-pyrolysis under 20 wt.% loading, we synthesized a series of clay mineral biochar composites and characterized their resultant properties and adsorption capacity.

#### 2.1.1. Adsorbent Surface Morphology

The specific surface area (S_BET_) of original biochar, single clay modified biochar, and magnetic clay modified biochar is 5.43, 249.33 and 264.03 m^2^/g, respectively, as shown in Table 1. After clay modification, the S_BET_ of BNT-BC has greatly increased, while the average pore diameter has decreased from 10.61 nm to 2.14 nm. It can be concluded that clay particles have successfully deposited on the surface of biochar, which is conducive to the increase in S_BET_ and the formation of microporous structure [37]. In contrast, the S_BET_ and pore size of the clay modified biochar impregnated with Fe(NO_3_)_3_·9H_2_O have not changed significantly, proving that the impregnation step of Fe(NO_3_)_3_·9H_2_O increases the magnetism of the mineral-loaded char without destroying its pore structure on the surface, which does not affect its capacity as an adsorbent.

**Figure 1:**
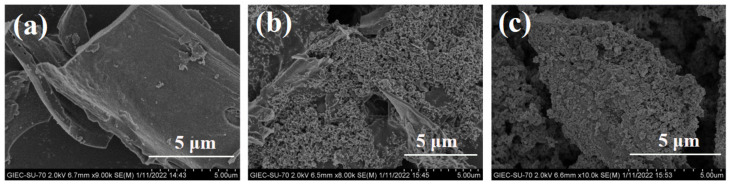
SEM images of BC (**a**), BNT-BC (**b**), and BNT-MBC (**c**).

Figure 1 shows the SEM images of the prepared adsorbents, and Table 2 lists their element composition analysis, comparing the surface morphology and surface element content of the biochar before and after modification. It indicates the presence of an inconspicuous pore structure on the surface of BC, and the elements composition analysis shows that on its surface were C and O, with a few K and Si (Figure 1a). Since biochar is derived from agricultural wastes, the surface morphology described here is random and disordered, which is due to the heterogeneity of biomass for pyrolysis. Pyrolysis under high temperature is conducive to volatilization of volatile compounds and formation of aromatic carbon structure, thus providing more sites for modification [38].

The SEM images agreed with the results of the nitrogen adsorption and desorption analysis described above. After clay modification and Fe (NO_3_)_3_·9H_2_O impregnation (BNT-MBC), dense spherical particle structure with uniform distribution appeared on the surface of biochar, with no large-scale agglomeration, proving that biochar is a good matrix for dispersing nano-scale γ-Fe_2_O_3_ and clay particles. The modification gave the surface of biochar a rougher appearance as well as providing a larger surface area for reactions. Enhanced porosity was observed on the surface of biochar, indicating that the adsorption of Cd(II) and MO could be supported by physical adsorption mechanisms such as increasing surface area and pore filling. The content of Fe has increased significantly after modification. In addition, the content of Al and Si, the typical element composition of clay minerals, has also been greatly increased.

#### 2.1.2. The FTIR Spectrum Analysis

**Figure 2:**
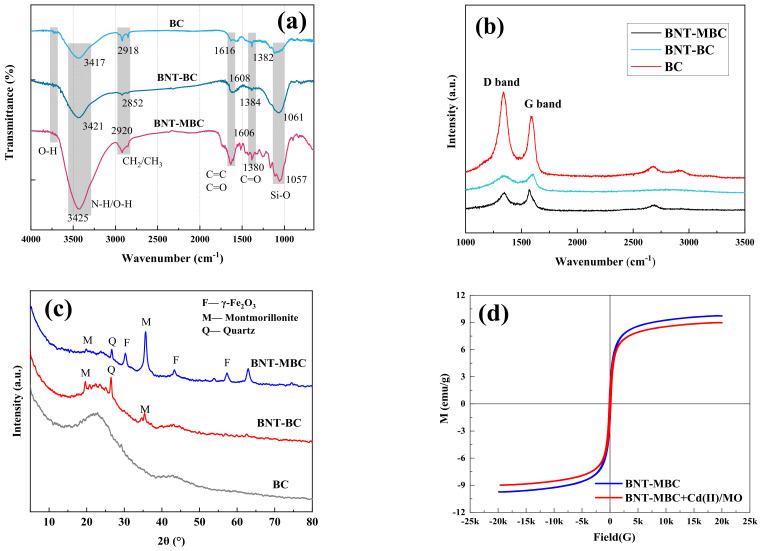
(**a**) FTIR spectra of BC, BNT-BC, and BNT-MBC; (**b**) Raman spectra of BC and BNT-MBC; (**c**) XRD of biochar of BC, BNT-BC, and BNT-MBC; (**d**) Hysteresis loop of BNT-MBC before and after adsorption.

The FTIR spectrum is shown in Figure 2a, which reflects the kind and intensity of the functional groups on the surface of adsorbents. The peak appearing at 1430 m^−1^ is attributed to C=O of carboxyl group [39]. The peak of 1650–1500 cm^−1^ is related to the vibration of C=C and C=O, which is attributed to the stretching aromatic ring produced during pyrolysis. The peak of 2900 cm^−1^ might be due to the vibration of a methyl group. The peak of 3500–3300 cm^−1^ is related to the vibration of -OH. Compared with the unmodified biochar BC, the -OH peak intensity of clay modified biochar has obviously increased. The order of hydroxyl peak intensity in the figure is BNT-MBC > BNT-BC > BC, indicating that clay and Fe (NO_3_)_3_·9H_2_O modification is conducive to increasing the surface functional group intensity of biochar. In addition, the broadening of the BNT-MBC peak at this site might be due to the superposition of vibrations between the -OH bonds of bentonite and BC after co-pyrolysis [40]. The modified biochar has a significantly enhanced peak around 1040–1080 cm^−1^, corresponding to the enhanced content of surface Si-O functional groups [41], which is the signature structure of clay-like compounds, implying that clay has been effectively loaded on the biochar surface.

#### 2.1.3. The Raman Analysis

In the Raman spectra, the D peak around 1300 cm^−1^ is relevant to the sp^3^ vibration of disordered carbon, representing defects in the lattice of carbon atoms. The peak at 1580 cm^−1^ is the characteristic absorption peak of the G peak, resulted from the in-plane sp^2^ mixed C-C bond stretching vibration, denoting its intact graphitized structure. The ratio of the D and G peak intensities, I_D_/I_G_, is an important means of determining the degree of graphitization for carbon materials. The smaller I_D_/I_G_ corresponds to a lower disorder degree and a higher graphitization degree. As shown in Figure 2b, the I_D_/I_G_ ratio of BNT-MBC and BNT-BC are not significantly different, but both are lower than BC, indicating that the clay modification and Fe(NO_3_)_3_·9H_2_O modification improve the graphitization to some extent [42]. The closer the structural properties of biochar are to the graphite layer structure, the easier it is to bonding stronger π-π conjugate structural bodies with the benzene rings and similar structures, which is more favorable for the adsorption of organic pollutants such as MO.

#### 2.1.4. The XRD Analysis

The XRD pattern exploited the phenomenon of X-ray diffraction in crystals to explore the physical phase information and crystal structure. In Figure 2c, the peaks of biochar at 2θ = 24.8° and 43.9° indicates the presence of amorphous carbon [43]. The peaks of BNT-MBC at 2θ = 30.1°, 43.1°, and 57.2° correspond to (220), (311), (400), and (531) crystallographic planes, the average crystallite size of which is 226 Å, with a relatively low crystallinity [44]. It can be speculated that Fe (NO_3_)_3_·9H_2_O loaded on biochar was pyrolyzed to form solid carbonaceous particles, where γ-Fe_2_O_3_ was formed after a series of dehydration. The peaks of BNT-MBC at 2θ = 19.9°, 26.5°, 35.3° correspond to the main bentonite crystal structure, indicating that clay particles were loaded on the biochar surface [45].

#### 2.1.5. Magnetic Properties of Modified Biochar

Adding magnetism to the adsorbent allows it to be easily separated from the solution by a magnet after use. The magnetic properties of BNT-MBC were investigated by hysteresis loops. Its saturation magnetization before adsorption reaches 9.71 emu/g, which ensures that this adsorbent can be easily separated by magnet. Magnetic properties of BNT-MBC can be used to separate the adsorbent from the solution, thus saving cost and mitigating secondary contamination. In addition, the magnetic loss of BNT-MBC after adsorption is relatively low, indicating that the magnetic properties of biochar can also remain stable after the adsorption and desorption process, which is conducive to the long-term recycling of the adsorbent.

### 2.2. Adsorption Experiments

#### 2.2.1. Adsorption under Different Preparation Conditions

**Figure 3:**
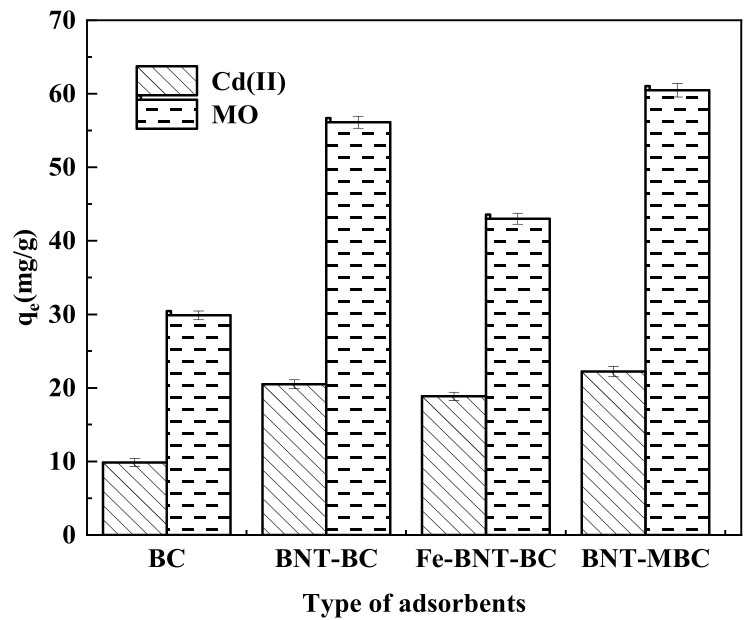
Adsorption capacity of Cd(II) and MO by different types of adsorbents (adsorbent dose 20 mg, contaminant solution 10 mL, pH 6, constant temperature 25 °C, and reaction time 24 h).

To investigate the optimal preparation conditions for synthesizing magnetic clay biochar synthetic materials with best adsorption effect, we compared the adsorption capacity of BC, BNT-BC, Fe-BNT-BC, and BNT-MBC, and the results are shown in Figure 3. The modified adsorbent obtained under the three preparation conditions of single clay co-pyrolysis, secondary pyrolysis of modified clay impregnated with Fe (NO_3_)_3_·9H_2_O, and pyrolysis of clay suspension containing iron ions after impregnation all exhibited better adsorption effect than the original biochar. Whether the adsorbate was Cd(II) or MO, BNT-MBC had the best adsorption effect among all. Biomass raw materials were impregnated in clay suspension containing iron ions and then pyrolyzed, which does not affect their adsorption capacity or even improve it while adding magnetism. It is speculated that the iron oxide formed on the surface of biomass raw materials after pyrolysis would complex with pollutants [36]. In the preparation of Fe-BNT-BC, Fe (NO_3_)_3_·9H_2_O might not impregnate the BNT-BC obtained from pyrolysis as well as biomass raw materials. Therefore, BNT-MBC was used as adsorbent in subsequent adsorption fitting experiments.

#### 2.2.2. Adsorption Isotherms 

**Figure 4:**
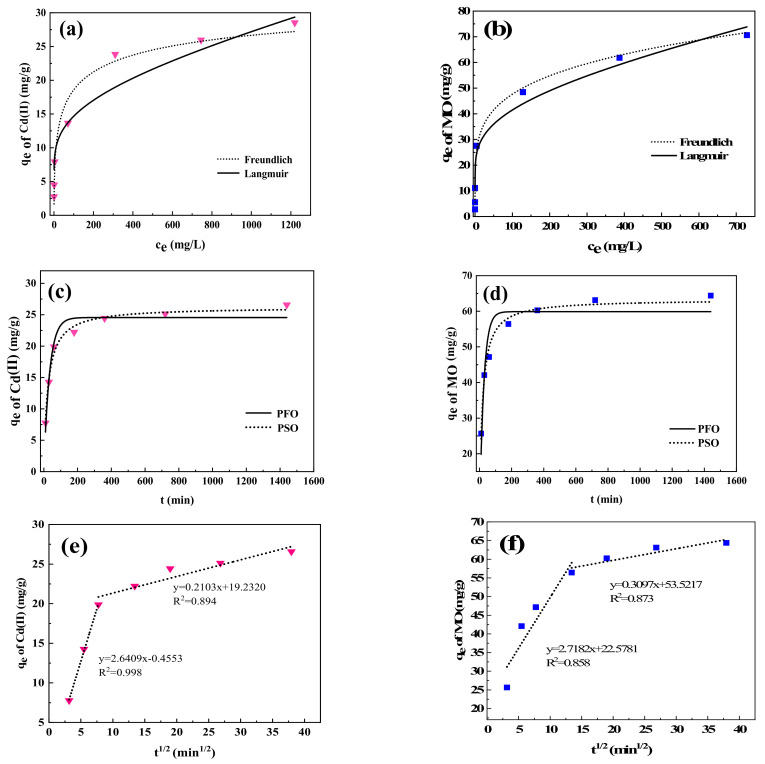
Fitting of adsorption isotherms of Cd(II) (**a**) and MO (**b**); Fitting of adsorption kinetics of Cd(II) (**c**) and MO (**d**); Fitting of intra-particle diffusion models of Cd(II) (**e**) and MO (**f**) by BNT-MBC (adsorbent dose 20 mg, contaminant solution 10 mL, pH 6, constant temperature 25 °C, and reaction time 24 h).

As seen in Figure 4a,b, the adsorption processes of Cd(II) and MO with BNT-MBC were fitted with isotherm models of Langmuir and Freundlich, respectively. For Cd(II) adsorption, the correlation coefficient (R^2^) of the Freundlich model (0.98445) was greater than that of Langmuir model (0. 86204) as shown in Table 3, indicating the presence of multilayer adsorption, the adsorption curve of which is nonlinear curve [46,47]. This suggests that this adsorption process biochar can physically interact with Cd(II) through pore-filling mechanism and intercalation [32]. Cd(II) reaches the interlayer space of clay minerals through exchange with hydrated cations (Na^+^, Ca^2+^).

However, the adsorption process of MO shows a good correlation with both Freundlich model (R^2^ = 0.99132) and Langmuir model (R^2^ = 0.94847), indicating there are both single-layer and multi-layer adsorption [48]. In addition to the aforementioned pore-filling mechanism, chemical adsorption such as π-π interaction and hydrogen bond also occurs in the adsorption process of MO at the same time.

#### 2.2.3. Adsorption Kinetics 

To explore the adsorption mechanism of BNT-MBC on Cd(II) and MO, PFO and PSO adsorption kinetics models were applied. The adsorption capacity increased rapidly in the initial stage, and the adsorption sites were gradually occupied with increasing contact time, followed by a slow increase to reach saturation within 4 h, as can be seen from Figure 4c,d. The adsorption processes of either Cd(II) or MO fit the PSO model better, with correlation coefficients R^2^ of 0.98733 and 0.98181, respectively, both of which are greater than their corresponding PFO models as shown in Table 4. It can be seen that the adsorption processes of both BNT-MBC for Cd(II) and MO are related to the active adsorption sites on surface, which are dominated by chemisorption. The rate-limiting step of both adsorbates can be a chemisorption process, which may be related to electron sharing or exchange between the adsorbate and the adsorbent.

To investigate the main limitation in the adsorption process, an intra-particle diffusion model was applied to these data as shown in Figure 4e,f. It demonstrated a multiple linear relationship between q_e_ and t^1/2^ in the BNT-MBC adsorption process, which could be used to investigate whether there was external mass transfer and intra-particle diffusion in the adsorption process, or only one of them [49]. In this study, it can be seen that the data of adsorbing Cd(II) and MO can be well-fitted by two straight lines, indicating that the adsorption process is a complex process controlled by two steps. The initial stage is relevant with the diffusion of adsorbate molecules from the solution to the outer surface of BNT-MBC. In the subsequent stages, the diffusion rate decreased, and the adsorbate entered the active site inside the modified biochar. None of the straight lines went through the origin, which proved that the internal diffusion of particles is not the only rate-limiting step during the adsorption process.

#### 2.2.4. Experiment of Desorption–Adsorption Cycle

**Figure 5:**
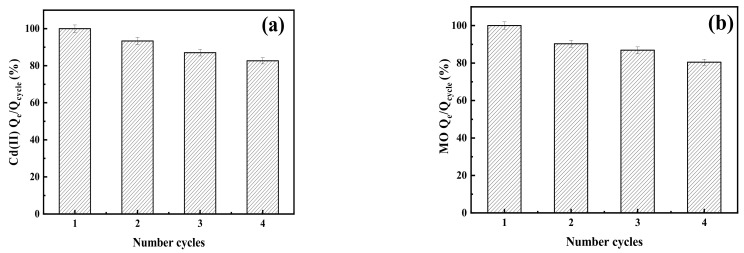
Experiment of desorption–adsorption cycle of BNT-MBC on (**a**) Cd(II) and (**b**) MO.

The reusability of the adsorbent is also a point of interest in the performance of the adsorbent. Figure 5a shows the adsorption capacities of BNT-MBC for Cd(II) under four cycles, with 1 M CH_3_COONa for desorption between cycles. In the experiment of desorption–adsorption cycle of BNT-MBC on Cd(II) and MO, the first cycle was set as the control group, and the adsorption capacity in the first cycle is regarded as saturated adsorption capacity (100%). The removal efficiency of Cd(II) by BNT-MBC reached 93.36% after the first adsorption–desorption cycle, and gradually decreased to 82.66% after three cycles, approximately 21.5 mg/g. The removal capacity of BNT-MBC for MO after four cycles is shown in Figure 5b. After four adsorption–desorption cycles, the adsorption capacity of MO is still higher than 80%. The decline of adsorption capacity may be caused by the destruction of the surface structure of biochar and the loss of active mineral composition [50]. 

### 2.3. Study on Cd(II)-MO Binary System Adsorption

**Figure 6:**
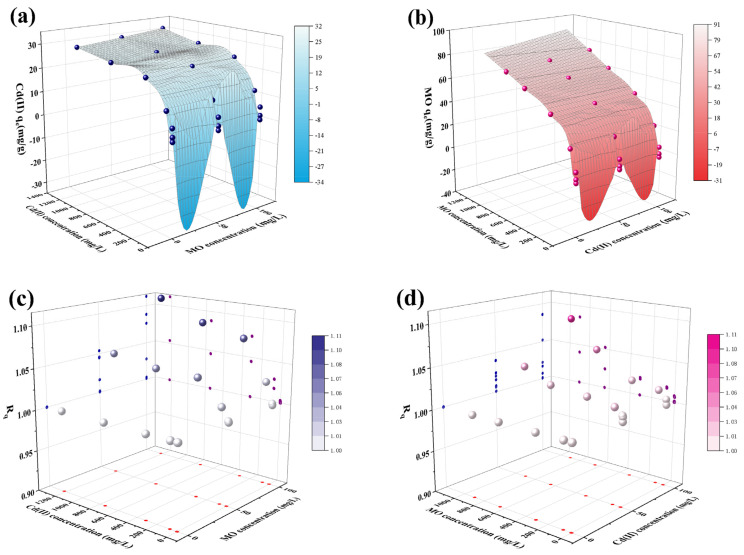
The adsorption of Cd(II) and MO in binary system by BNT-MBC: the influence of different initial concentrations on adsorption capacity (**a**) and R_q_ (**c**) of Cd(II), on adsorption capacity (**b**) and R_q_ (**d**) of MO (adsorbent dose 20 mg, contaminant solution 10 mL, pH 6, constant temperature 25 °C, and reaction time 24 h).

The adsorption effect was compared by adding 50 mg/L and 100 mg/L of MO or Cd(II) solution to the original single pollutant system to form three series of concentration gradients. Batch adsorption experiments were carried out using BNT-MBC as adsorbent in the binary system of Cd(II)-MO, the results of which are demonstrated in Figure 6. For comparing the adsorption effect in the single pollutant and binary systems more clearly, the adsorption capacity ratio *R_q_* was introduced to evaluate the interaction between the pollutants, as shown in Equation (1).

(1) $$R_{q}=\frac{q_{b,i}}{q_{m,i}}$$

where *q_m,i_* and *q_b,i_* are the absorption capacities of pollutant *i* in the single pollutants and binary systems with same initial concentration. If *R_q_* > 1, it is proven that the pollutants in the environment have a positive impact on the adsorption of adsorbate *i*, which promotes its adsorption process; when it is equal to 1, it indicates that the two coexisting pollutants have no influence on each other; if it is less than 1, it indicates that inhibition may occur [51].

Figure 6a shows that as the concentration of added MO increases, the adsorption capacity of BNT-MBC on Cd(II) also increases; the adsorption process on MO in Figure 6b also shows the same pattern. Figure 6c,d shows that whether Cd(II) or MO is the main adsorbate, the adsorption capacity ratio *R_q_* in the mixed system is greater than one, indicating that another pollutant coexisting in the solution can promote the adsorption of the original adsorbate to varying degrees. 

It can be inferred that after BNT-MBC adsorbs MO, the -SO_3_^−^ group of MO will be exposed, providing more active adsorption sites for Cd(II), thus increasing the adsorption amount of Cd(II). In addition, the surface electronegativity of the adsorbent with negative surface is stronger than before after absorbing the anionic dye MO, and it is easier to adsorb Cd(II) by electrostatic attraction. At the same time, it is also proven that Cd(II) and MO do not occupy each other’s active adsorption sites on BNT-MBC surface in the adsorption process, and there is no competitive relationship in the adsorption process.

### 2.4. Co-Adsorption Mechanisms Analysis

It can be concluded from the above characterization and fitting results, the clay particles were successfully deposited on the surface of adsorbent, which is conducive to the enhancement of specific surface area and the formation of microporous structure. After the clay particles were successfully loaded on the adsorbent surface, cationic pollutants in wastewater could exchange with hydrated cations (such as Na^+^ and Ca^2+^) inside the clay particles and reach the interlayer space of the clay minerals [32,41]. In addition, the modification enhanced oxygen-containing functional groups on adsorbent surface, such as the peak appearing at 1430 m^−1^ corresponding to carboxyl group and the peak of 3500–3300 cm^−1^ corresponding to the –OH stretching, which leads to the easier occurrence of surface complexation reaction with HMs [52]. The analysis of Raman spectra also exhibited that BNT-MBC has a higher degree of graphitization. Previous studies have shown that the combination with oxygen-containing functional groups and electron-rich domains on graphene-like structure might be the main mechanism for adsorption of Cd(II) by adsorbents with low cation exchange capacity, and π-π conjugated structure and benzene ring structure are also more conducive to adsorption of organic pollutants [53,54,55].

**Figure 7:**
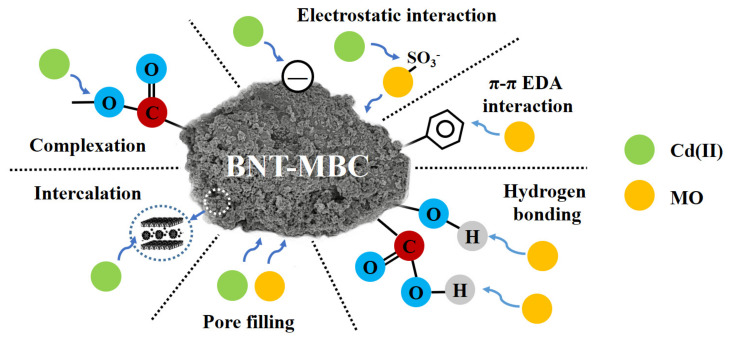
Hypothesis mechanism of co-adsorption of MO and Cd(II) by bentonite−biochar composite.

With the analysis and discussion of the above experimental results combined, the main adsorption mechanism of BNT-MBC was summarized as can be seen in Figure 7. For Cd(II) adsorption, there are physical adsorption processes such as pore filling and intercalation, which correspond to the fitting results of its multilayer adsorption; Cd(II) also adsorbs on the negatively charged adsorption sites on the surface of clay-biochar composite material through electrostatic interactions; the complexation reaction between Cd(II) and oxygen-containing functional groups on the surface of carbon materials also exists. For MO adsorption, MO can be adsorbed on the BNT-MBC surface through hydrogen bonding, π-π EDA interaction, and electrostatic interaction; MO can be bonded to O-H through hydrogen bonding as well as electrostatic interaction; the biochar surface as an electron donor forms a stronger π-π conjugated structural body with benzene ring and similar structures, which is more favorable for MO adsorption.

For systems in which heavy metals and organic pollutant dyes co-exist, Cd(II) and MO can contribute to the adsorption of another adsorbent to different degrees. The exposed -SO_3_^−^ group of MO adsorbed by BNT-MBC increases the adsorption sites of Cd(II); the enhanced surface electronegativity of BNT-MBC after adsorption of the anionic dye MO makes it easier to adsorb Cd(II) via electrostatic attraction. Cd(II) and MO do not compete with each other in the adsorption process and do not occupy each other’s active adsorption sites. Therefore, based on the results above, the magnetic clay biochar composite BNT-MBC is effective in simultaneously removing Cd(II) and MO contaminant from wastewater.

## 3. Materials and Methods

### 3.1. Materials and Reagents

Corn straw, as the raw biomass, was obtained from Guangdong, China. Bentonite, suitable for medium and high polar solvents, was purchased from Guangdong, China. In addition, the chemical reagents involved in the study, such as Cd (NO_3_)_2_·4H_2_O, MO, Fe (NO_3_)_3_·9H_2_O, CH_3_COONa, and some other reagents for pH adjustment were purchased from Shanghai Macklin Biochemical Co., Ltd. (Shanghai, China), all of which were analytical reagents.

### 3.2. Preparation of Magnetic Clay-Biochar Composites

The corn straw was washed and dried, ground and passed through a 120-mesh sieve. An amount of 10 g of ground corn straw was packed into a quartz boat, and then was pyrolyzed using a tube furnace under nitrogen atmosphere. Referring to other studies on the pyrolysis of biocarbon-based synthetics as adsorbents [30], the heating program was set at 10 °C/min, and the ground corn straw were pyrolyzed under 600 °C for 60 min, followed by continuous cooling in an inert atmosphere to prevent oxidation. The resulting sample was named as BC.

Bentonite (2 g) was added into 500 mL deionized (DI) water, and then homogenized via ultrasonic for 30 min to obtain a stable clay suspension. The ground corn straw (10 g) was immersed in the clay suspension above and stirred for 1 h, then separated and dried at 100 °C. The clay-treated corn straw was placed in a quartz tube in a tubular furnace, pyrolyzed at 600 °C for 1 h under the protection of nitrogen, where the heating speed was 10 °C/min. The obtained material was named as BNT-BC. 

The aforementioned BNT-BC and Fe (NO_3_)_3_·9H_2_O were mixed with a mass ratio of 1:1, then stirred magnetically for 2 h, finally dried to constant weight under 100 °C. The dried Fe (NO_3_)_3_·9H_2_O/BNT-BC mixture was placed in a tube furnace and pyrolyzed at 600 °C for 1 h under the nitrogen atmosphere. The obtained modified biochar was named as Fe-BNT-BC.

An amount of 2 g of bentonite and 10 g of Fe(NO_3_)_3_·9H_2_O were added into 500 mL deionized water, and then a stable clay suspension containing iron ions for the modification of biomass was obtained after ultrasonically treating for 30 min. An amount of 10 g of ground corn straw were immersed in this suspension and stirred for 1 h, then separated from the mixture and dried under 80 °C. The clay-treated biomass material was pyrolyzed in a tubular furnace under 600 °C for 1 h in N_2_ flow, and the obtained composite material was named as BNT-MBC.

### 3.3. Adsorbent Characterization

The BET (Brunauer, Emmett and Teller technique) surface area, pore size distribution and total pore volume of adsorbent were characterized with a surface area and porosimetry analyzer (Micromeritics ASAP 2460, Norcross, GA, USA). Scanning electron microscope (Hitachi Ultra High-Resolution Analytical FE-SEM SU-70, Tokyo, Japan) was used for surface morphology, and Fourier transform infrared spectrometer (Thermo Scientific™ Nicolet™ iN10, Waltham, MA, USA) was used to describe the type and content of functional groups of biochar. Physical phase analysis was conducted on an X-ray diffractometer (X’Pert Pro MPD, Heracles Almelo, The Netherlands) with a conventional angular (5 to 100°) physical phase scan at room temperature. A laser confocal Raman spectrometer (Horiba Jobin-Yvon, LabRAM HR800, Paris, France) was used to determine the molecular vibration spectra. The concentration of Cd(II) was analyzed using an inductively coupled plasma emission spectrometer (ICP-OES Optima 8000, PerkinElmer, Waltham, MA, USA) while the concentration of MO was measured with an ultraviolet spectrophotometer (Lambda750, PerkinElmer, Waltham, MA, USA) at the wavelength of 463 nm.

### 3.4. Adsorption Experiments

Several sets of adsorption experiments were implemented to figure out the adsorption capacity of clay biochar composites with different synthesis methods. We took 20 mg of the aforementioned BC, BNT-BC, Fe-BNT-BC, or BNT-MBC, adding them to a centrifuge tube (15 mL) along with 10 mL of contaminant solution (Cd(II) initial concentration of 1000 mg/L or MO of 500 mg/L) while controlling pH at 6 and shaking at a constant temperature of 25 °C for 24 h. Equilibrium concentration of Cd(II) after adsorption was measured with an ICP-OES, and the concentration of MO was measured using a UV spectrophotometer at 463 nm.

An amount of 1 M CH_3_COONa was used in the desorption experiment of biochar attached with Cd(II), while absolute ethanol was used as the desorption agent in the desorption experiment of MO, other experimental settings of which were consistent with other adsorption experiments.

In the binary adsorption experiment, the concentration gradient of Cd(II) solution was set at 10–2000 mg/L, and 50 or 100 mg/L MO solutions were added to compare the effects of different MO concentrations on adsorbing Cd(II). The concentration gradient of MO solution is set to 10–1000 mg/L, and Cd(II) solutions (50 mg/L and 100 mg/L) were also added. Cd(II) concentration was measured using an ICP-OES, while MO measured with a UV spectrophotometer under 463 nm.

### 3.5. Adsorption Data Analysis Methods

In the experiments of adsorption isotherm, the initial concentration gradient of Cd(II) solution was set at 10–2000 mg/L, and the concentration gradient of MO solution is set to 10–1000 mg/L. Other reaction conditions were kept consistent with the adsorption experiments (adsorbent dose 20 mg, contaminant solution 10 mL, pH 6, constant temperature 25 °C, and reaction time 24 h). The data of MO and Cd(II) adsorption on BNT-MBC composites were fitted using Langmuir and Freundlich adsorption isotherm model.

(2) $$\mathrm{Langmuir}\;\mathrm{isotherm}\;\mathrm{equations}:\;q_{e}=q_{m}K_{L}c_{e}\cdot {\left(1+K_{L}c_{e}\right)}^{-1}$$

(3) $$\mathrm{Freundlich}\;\mathrm{isotherm}\;\mathrm{equations}:q_{e}=k_{F}{c_{e}}^{n}$$

where, *q*_m_ (mg/g) is the maximum adsorption capacity for Langmuir; *k*_L_ (L/mg) is Langmuir constant; *c*_e_ (mg/L) is the concentration of pollutants at equilibrium; *k*_F_ (mg/g) is the corresponding adsorption capacity; *n* is Freundlich constant.

In the experiment of kinetic fitting, the reaction time intervals ranged from 0.5 h to 24 h, while other reaction conditions were kept consistent with the adsorption experiments (adsorbent dose 20 mg, contaminant solution 10 mL, pH 6, and constant temperature 25 °C). The data of MO and Cd(II) adsorption on BNT-MBC composites were fitted using pseudo first-order (PFO) and pseudo second-order model (PSO).

(4) $$\mathrm{PFO}\;\mathrm{kinetic}\;\mathrm{model}:\;q_{t}=q_{e}\left(1-e^{-k_{1}t}\right)$$

(5) $$\mathrm{PSO}\;\mathrm{kinetic}\;\mathrm{model}:\;q_{t}=k_{2}{q_{e}}^{2}t\cdot {\left(1+k_{2}q_{e}t\right)}^{-1}$$

where *k*_1_ (min^−1^) and *k*_2_ (g/(mg·min)) are the reaction rate constants of PFO and PSO; *t* (min) represents the reaction time; *q_t_* (mg/g) is the adsorption capacity in time; *q_e_* (mg/g) is the adsorption capacity at equilibrium.

## 4. Conclusions

In this study, a novel magnetic clay-modified biochar BNT-MBC was prepared by co-pyrolysis of bentonite and impregnation of Fe (NO_3_)_3_·9H_2_O. The adsorption effect of Cd(II) and MO is remarkable, the maximum adsorption capacity of which are 26.22 and 63.34 mg/g, respectively. As a kind of clay material with low cost and easy access, bentonite shows good modification characteristics, and the addition of magnetism makes it easier to separate and recover after adsorption. In addition, the adsorption mechanism of single pollutant system and binary pollutant system is also explored. The isotherm fitting results indicate that Cd(II) is adsorbed in multiple layers on the BNT-MBC surface, while MO is adsorbed in both monolayer and multilayer. The experimental results of Cd(II) and MO adsorption are consistent with the PSO model, and chemisorption is the rate-limiting step. The fitting results of intra-particle diffusion indicate that the adsorption process is a complex process controlled by two steps. In the binary system of Cd(II) and MO, the coexistence of another pollutant has a facilitating effect on adsorbing the original pollutant, and the active adsorption sites of Cd(II) and MO are different, while the electronegativity of the surface after adsorption of the anionic dye MO is more intensified, which makes it easier to adsorb Cd(II) via electrostatic attraction. BNT-MBC also shows good reusability for Cd(II) and MO removal, making it an environmentally friendly material. In addition, the desorbed Cd(II) and MO can be magnetically recovered for recycling. Based on the above results, the magnetic clay biochar composite BNT-MBC is an effective and economical material for simultaneous removing of Cd(II) and MO from wastewater.

## Figures and Tables

**Table 1:** Pore structure analysis of BC, BNT-BC, and BNT-MBC.

Adsorbent	S_BET_ (m^2^/g)	Micropore Area (m^2^/g)	V_tot_ (cm^3^/g)	Pore Width (nm)
BC	5.42	0.30	0.0144	10.61
BNT-BC	249.33	225.78	0.1294	2.05
BNT-MBC	264.03	227.46	0.1423	2.14

**Table 2:** Element composition of BC, BNT-BC, and BNT-MBC.

Adsorbents	Element Composition (%)					
	C	O	Fe	Al	Si	K
BC	83.82	14.02	-	-	0.31	0.74
BNT-BC	84.35	13.55	-	0.77	0.87	0.1
BNT-MBC	54.73	26.88	12.23	3.12	2.86	0.19

**Table 3:** Fitting parameters of adsorption isotherm eith BNT-MBC.

Adsorbate	Freundlich			Langmuir		
	k_F_	n	R^2^	q_m_ (mg/g)	k_L_	R^2^
Cd(II)	3.57146	0.54228	0.98445	33.49100	0.00148	0.86204
MO	19.83155	0.18022	0.99132	18.87579	1.00028	0.94847

**Table 4:** Fitting parameters of adsorption kinetics by BNT-MBC.

Adsorbate	PFO			PSO		
	q_e_ (mg/g)	k_1_	R^2^	q_e_ (mg/g)	k_2_	R^2^
Cd(II)	24.55506	0.0298	0.94869	26.22295	0.00162	0.98733
MO	59.88354	0.04021	0.86677	63.33823	9.76543	0.98181

## Data Availability

No applicable.
